# Plasma Tryptophan/Large Neutral Amino Acids Ratio in Domestic Dogs Is Affected by a Single Meal with High Carbohydrates Level

**DOI:** 10.3390/ani8050063

**Published:** 2018-04-25

**Authors:** Angelo Gazzano, Asahi Ogi, Beatrice Torracca, Chiara Mariti, Lucia Casini

**Affiliations:** Department of Veterinary Science, University of Pisa, 56124 Pisa, Italy; angelo.gazzano@unipi.it (A.G.); beatrice.torracca@vet.unipi.it (B.T.); chiara.mariti@unipi.it (C.M.); lucia.casini@unipi.it (L.C.)

**Keywords:** l-tryptophan, dog, large neutral amino acids, diet

## Abstract

**Simple Summary:**

Many studies have reported that aggressive dogs have low serotonin plasmatic levels. l-tryptophan (TRP) is the precursor of serotonin and its availability is affected by five large neutral amino acids (isoleucine + leucine + phenylalanine + tyrosine + valine) (5LNAAs). The passage of TRP through the blood–brain barrier uses carriers, where it competes with 5LNAAs. Hence, a diet modification could be useful to alter their plasma ratio, in improving TRP bioavailability. Five female Labrador Retrievers were fed three different meals: M1 (a mix of puffed rice, minced meat and olive oil), M2 (puffed rice and olive oil) and M3 (commercial dry food usually consumed). Blood was collected right before the first meal (t0) and after 2, 4, 6, 8, 10 and 24 h. Plasmatic TRP concentrations showed no significant difference between M1, M2 and M3 samples. M2 led to a decrease in 5LNAAs levels and consequently led to significant higher TRP/5LNAAs ratios in the 6 h period after the provision of carbohydrates, compared to both M1 and M3. In addition, the mean TRP/5LNAAs ratio was significantly higher in M2 than in M3, at t8 and t10. These results indicate that meal composition affects TRP/5LNAAs ratio and possibly, TRP bioavailability.

**Abstract:**

Aim of this study was to evaluate the plasma ratio between l-tryptophan (TRP) and five large neutral amino acids (isoleucine + leucine + phenylalanine + tyrosine + valine) (5LNAAs) after a single meal with high carbohydrates level. Five female Labrador Retrievers were involved. Each dog was fed three different meals: M1 (a mix of puffed rice, minced meat and olive oil), M2 (puffed rice and olive oil) and M3 (commercial dry food usually consumed) once in the morning for one single day every 30 days. Blood was collected right before the first meal (t0) and after 2, 4, 6, 8, 10 and 24 h. Plasma amino acids’ concentrations were measured using an HPLC (High-performance liquid chromatography) method with fluorimetric detection. Plasmatic TRP concentrations showed no significant difference between M1, M2 and M3 samples at any sampling time. M2 led to a decrease in 5LNAAs levels and consequently led to a significant higher TRP/5LNAAs ratios in the 6 h period after the provision of carbohydrates, compared to both M1 and M3. In addition, the mean TRP/5LNAAs ratio was significantly higher in M2 than in M3 at t8 and t10. These results indicate that meal composition affects TRP/5LNAAs ratio and possibly, TRP bioavailability.

## 1. Introduction

Dogs have shared their lives with humans for at least 15,000 years [1] and they can develop a strong attachment bond with humans [2]. However, this relationship is sometimes negatively influenced by the onset of behavioral problems that may force the owner to relinquish the dog to a shelter [3]. Intra and interspecific aggression, phobias and separation problems are the most frequent behavioral causes of relinquishment [3]. These problems can result from many causes, including an altered functionality of serotoninergic pathways in the brain, characterized by serotonin (5-HT) deficiency [4,5,6]. 

The serotoninergic system establishes a connection between the subcortical centers (where vital functions and emotional life are regulated), peripheral nervous system and cortex, and it modulates a wide range of physiologic functions [7]. Many of the currently prescribed therapies involve the use of drugs that selectively inhibit 5-HT reuptake at the synaptic level, thereby increasing its availability [4,5,6].

However, sometimes veterinary behaviorists find difficult to administer such therapies. In some countries, these drugs are not registered for dog use and they are very expensive, especially for long-term therapies. Moreover, dog owners sometimes disagree to administering drug therapy to their pets for ethical or personal reasons. It is therefore interesting to evaluate whether 5-HT concentrations can be increased differently, i.e., by increasing the availability of its precursor l-tryptophan (TRP), of which only 1% is converted to 5-HT [8].

The synthesis and release of 5-HT by brain neurons are in fact rapidly influenced by the local TRP concentration [9,10]. Due to the TRP bound to albumin in blood for 70–90% [11] at the cerebral microcirculation level, the dissociation of TRP from albumin [12] is necessary to overcome the blood–brain barrier. TRP may be displaced from this bond by drugs (salicylates, chlorpromazine) [13], as well as by other substances such as non-esterified fatty acids [14] and bilirubin [15]. The passage of TRP through the blood–brain barrier uses carriers for which it competes with other large neutral amino acids (LNAAs), mainly leucine, isoleucine, valine, tyrosine and phenylalanine [16].

In the brain, TRP is hydroxylated to 5-hydroxytryptophan by the enzyme TRP hydroxylase, that is usually about half saturated with the amino acid [17]. The 5-hydroxytryptophan is then decarboxylated to 5-HT, which is stored in vesicles in the nerve terminal where it is held before it is released. Therefore, increases in TRP in the brain can maximally double the 5-HT synthesis [17].

The ingestion of food is one of the most effective physiological processes that alter the blood concentrations of TRP and its competitors (LNAAs). Consequently, it can modify the TRP content in the brain [18]. Previous studies have shown that the ingestion of a large carbohydrate, protein-free meal by fasting rats, rapidly raises brain TRP concentrations and stimulates 5-HT synthesis [19]. Meals containing protein fail to raise brain TRP concentrations because their ingestion causes the increase of both serum TRP and LNAA concentrations by proportionally similar amounts, resulting in no net change in competition for uptake [19].

The carbohydrate meal is known to produce its effects on TRP via insulin secretion, which depresses the plasma concentrations of LNAAs in mammals, increasing their uptake into muscle, except TRP due to its bond with albumin [20,21].

Diet manipulation can, therefore, hypothetically be used to produce a rise in plasma and, consequently, intracerebral TRP concentrations, leading to the synthesis and release of a greater amount of 5-HT. This may have positive effects on behavioral problems resulting from an altered function of serotoninergic systems. Previous studies reported some positive actions of a low-protein diet on dog behavior, but evidence of a real effect of this type of diet on the TRP bioavailability is, until now, missing.

De Napoli et al. [22] found that the addition of TRP to high protein diets or the switch to a low protein diet might reduce aggression in dogs displaying so-called dominance and territorial aggression. Plasma concentrations of 5-HT and TRP had consistent results in all phases of the study, despite different concentrations of dietary TRP. As suggested by the authors, this is most likely due to their inadequate analytic methods. Mugford [23] reported that there was a reduction in aggressive behaviors in three of the seven aggressive Golden Retrievers after the introduction of a low-protein diet (15–18% of total energy). However, the composition of the experimental and previous diets was not reported. In another study [24], twelve dogs that exhibited either high territorial aggression, dominance aggression or hyperactivity, and fourteen control dogs were fed each of three diets varying in protein content (180, 250 and 310 g crude protein/kg DM) for two weeks at living in-home situations. The low-protein diet and medium-protein diet decreased territorial aggression scores in comparison to the high-protein diet. Gatta et al. [25] did not find a statistically significant increase of reactivity at sudden stimuli in dogs fed a diet with 23.1% of carbohydrates, compared with a diet with a 46.9% carbohydrate content and the same protein level (29%). However, no TRP concentrations and TRP/5LNAAs were evaluated.

No effect of a diet with TRP addiction was noted by Bosch et al. [26] on the behavior of mildly anxious dogs. The intake of the TRP supplemented diet significantly increased plasma TRP concentrations by 37.4%, and its ratio with large neutral amino acids by 31.2% compared to the control diet. However, the owners did not report any behavioral changes that could be attributed to specific dietary treatment. The dogs’ responses to the behavioral tests, including their levels of salivary cortisol, were unaffected after eight weeks of consuming the TRP supplemented food.

In light of these contrasting results, it is interesting to test the bioavailability of TRP following the administration of a diet in which there is a temporal dissociation between main protein and carbohydrate sources. It is therefore conceivable that, even in dogs, a meal consisting exclusively of carbohydrates could alter the TRP/5LNAAs ratio in favor of TRP.

To test this hypothesis, the aim of this research was to evaluate the plasma ratio between TRP and 5LNAAs (isoleucine + leucine + phenylalanine + tyrosine + valine) after three different meals in dogs, each administered for a single day.

## 2. Materials and Methods

The study was approved by the Ethical Committee of the University of Pisa, Italy (protocol n° 38/16) in accordance with Directive 2010/63/EU.

Five female Labrador Retrievers (2 spayed), 8.6 ± 3.8 years old and 30.2 ± 2.3 kg BW (mean ± S.D.), from the same bloodline, were involved in the study. The dogs were in good health and nutrition state, with fasting glycemic values within the physiological range. Each dog was kept in its own house. For the current research, two different diets were administered for a single day: a homemade diet (D1) consisting of beef meat (about 63–64%), puffed rice (about 34–35%), and enough olive oil to balance the energy requirement and a commercial diet (D2). The energy requirement was evaluated according to NRC [27]. Diets were iso-energetic and iso-nitrogenous (Table 1).

In the day of the experiment, dogs were fed two meals: one at 8:00 a.m. and one after 12 h (8:00 p.m.). The plasma level of l-tryptophan (TRP) and 5LNAAs (isoleucine + leucine + phenylalanine + tyrosine + valine) were evaluated after the morning of administration (8:00 a.m.).

The ingredients of the different meals, each administered after 30 days interval, are reported in Table 2. The evening meal provided the daily amount of energy intake and was balanced with beef meat and the protein requirements for Meal 2 (M2). Meal 1 (M1) and M2 are simply a different administration of D1, in a mixed (M1) and dissociate form (M2).

After the diets administration tests, the dogs returned to their usual diet (D2), the same utilized for Meal 3 (M3).

Blood was collected right before the morning meal (t0) and after 2, 4, 6, 8 and 10 h. A further blood sampling was collected at t24 to evaluate the return to the basal value. Blood samples were drawn from the cephalic vein in heparinized tubes and were immediately centrifuged at 2000 *g* for 15 min at +5 °C in a refrigerated centrifuge (ALC4237R, ALC International Srl, Milan, Italy). Plasma was collected and stored at −20 °C for further processing.

The extraction and quantification of amino acids in plasma samples were performed following an HPLC method, as previously described in the literature [28,29,30] and based on precolumn derivatization with *o*-phthaldialdehyde (OPA) and fluorimetric detection. This method was slightly modified as follows: 100 µL of HClO_4_ 1.5 M were added to 100 µL of plasma sample or standard solution; the extract was then neutralized with 50 µL of K_2_CO_3_ 2 M and 800 µL of water, mixed, and centrifuged at 5000× *g* for 5 min.

The reagent solution was prepared fresh each day of analysis adding to 30 mg of OPA 0.75 mL of MeOH, 6.7 mL of sodium borate buffer (40 mM, pH 9.5), 30 µL of 2-mercaptoethanol and 240 µL of Brij35 solution (30% *w*/*v*). For the derivatization 100 µL of sample extract were added to 100 µL of OPA reagent and left to react for 60 s, and then used for HPLC injection.

HPLC analyses were performed using a RP Gemini C18 column (250 mm × 4.6 mm, 5 µm) (Phenomenex, Torrance, CA, USA) and a Jasco HPLC apparatus (Jasco Corporation, Ishikawa-machi Hachioji-shi, Tokyo, Japan) equipped with 2 gradient pumps (PU-1580), a mixer unit (HG-2080-03) and a fluorescence detector (FP-920).

The mobile phase consisted of MeOH (solvent A) and sodium acetate 0.1 M, 0.5% THF, 9% MeOH, pH 7.2 (solvent B) eluted at a flow rate of 1.1 mL/min. The elution gradient was as follows: time 0 min, 30% A; 10 min, 45% A; 24 min, 65% A; 25 min, 100% A, 30 min 100% A. Injection volume was 20 µL and the fluorescence detector was set at 340 nm excitation wavelength and 455 nm emission wavelength. Data was acquired using Jasco Borwin 1.5.0 software (Jasco Corporation, Ishikawa-machi Hachioji-shi, Tokyo, Japan).

l-isoleucine (ILE), l-leucine (LEU), l-phenylalanine (PHE), l-tryptophan (TRP), l-tyrosine (TYR) disodium salt hydrate, l-valine (VAL), *o*-phthalaldehyde (OPA), 2-mercaptoethanol, and sodium tetraborate decahydrate were purchased from Sigma-Aldrich Inc. (Saint Louis, MO, USA).

HPLC grade methanol (MeOH), hydrochloric acid (HCl), and potassium carbonate (K_2_CO_3_) were acquired from Carlo Erba Reagenti Spa (Milan, Italy). Perchloric acid (HClO_4_) and tetrahydrofuran (THF) were purchased from J.T. Backer Chemical (Center Valley, PA, USA).

Ultra pure water from a Millipore Milli-Q system (Millipore, Milan, Italy) was used for all the solutions.

Stock solutions (5000 µg/mL) of each amino acid were stored at −20 °C and used to prepare working solutions (ranging from 50 µg/mL to 0.1 µg/mL). It was also employed to identify chromatographic peaks and to calculate calibration curves.

All statistical analyses were performed with the software R^®^ ver. 3.3.1 (R Core Team, R Foundation for Statistical Computing, Vienna, Austria, 2016). TRP concentrations and the ratio between TRP and the sum of 5LNAAs (TRP/5LNAAs) at different time points of the three considered meals were compared using a mixed model for repeated measures (*p* < 0.05).

## 3. Results

**Figure 1:**
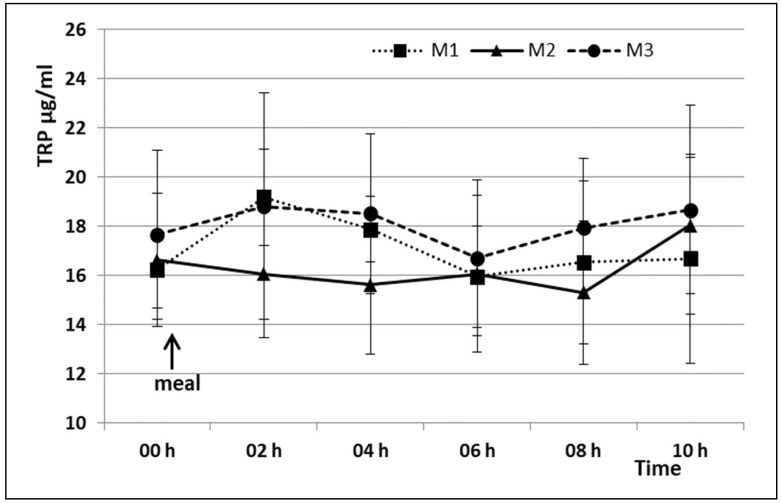
Mean plasmatic TRP concentrations (±S.D.) after three different meals. M1 = Meal 1; M2 = Meal 2; M3 = Meal 3.

TRP concentrations showed no significant difference among M1, M2 and M3 at any sampling times (Figure 1).

**Figure 2:**
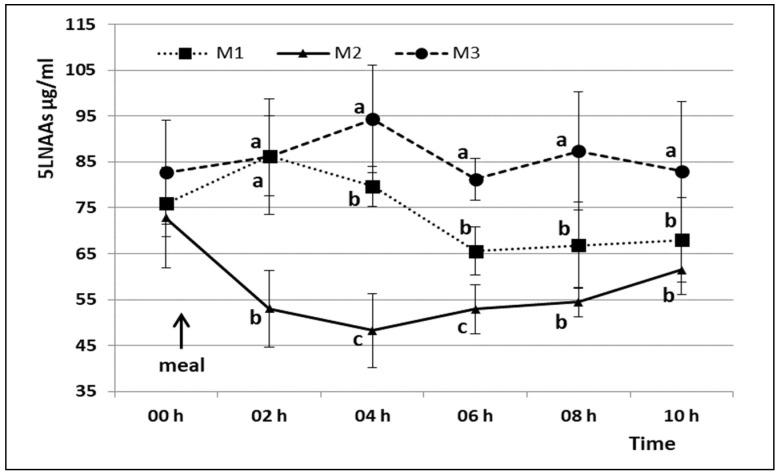
Mean 5LNAAs plasma concentration (±S.D.) at different times in the three administered diets. Different lowercase letters indicate statistically significant differences between diets at the same time point (*p* < 0.05). M1 = Meal 1; M2 = Meal 2; M3 = Meal 3.

The 5LNAAs levels (Figure 2) were similar at t0 in the three experimental days (75.89 ± 7.27, 72.84 ± 11.01 and 82.69 ± 11.37 µg/mL in M1, M2 and M3 respectively), but they showed different trends depending on the composition of the meal provided. 

In detail, M2 led to a significant decrease in 5LNAAs levels resulting in a higher TRP/5LNAAs ratios in the 6 h period after the provision of carbohydrates (Table 3).

## 4. Discussion

In extending from a prior similar study on rats [18], to our knowledge, the results of this preliminary study present the first data about the bioavailability of the plasma TRP in the dog after three meals, each administered for a single day, with different percentages of proteins and carbohydrates.

In our study, the meals provided with the commercial diet (M3) and the mixed homemade diet (M1) showed no differences in the TRP/5LNAAs ratio, along with a 6 h period. Nevertheless, the same homemade diet showed a significant different trend for both 5LNAAs and TRP/5LNAAs ratio, in comparison with M1 and M3 when carbohydrate meal (M2) and protein sources were administrated separately.

Data suggests that M2 can significantly modify the TRP/5LNAAs ratio, keeping it higher than other meals for at least 6 h. The reduction of plasmatic 5LNAAs concentration has a well-known physiological explication in which increased glycaemia, induced by the carbohydrates intake, stimulates pancreas releases insulin, that is responsible for the observed changes [20]. Insulin, in fact, facilitates the intake of 5LNAAs into muscle fibers, reducing their availability, while it does not affect TRP level due to its binding to albumin [21].

Our findings might have a strong impact because the synthesis and release of 5-HT by brain neurons are rapidly influenced by the local TRP availability [9,10], which is also highly correlated to the decrease of 5LNAAs. Such condition could improve the TRP uptake throughout the blood–brain barrier.

An increase in TRP/5LNAAs ratio in plasma could influence the 5-HT levels in the brain and, consequently, could have a positive effect on some dog behavioral problems, characterized by a deficit of this neurotransmitter. For instance, 5-HT deficit seems to be involved in aggressive behavior in dogs and other species [5,6]. In-depth, Rosado et al. [5] found that aggressive dogs showed significantly lower serum concentrations of 5-HT than non-aggressive dogs; the lowest 5-HT concentrations were found in the group of dogs showing defensive forms of aggression. In addition, aggressive English Cocker Spaniels had significantly lower levels of serum 5-HT than aggressive dogs of other breeds [31].

Further studies are required to verify if the trend of TRP/5LNAAs ratio, observed after a single meal of carbohydrates, is also maintained during a continuous and prolonged administration of this diet, and if it is observed in the rat [19], whether a greater transport of TRP through the blood–brain barrier exists. The increase of TRP bioavailability during the day could, in fact, be applicable in the management of behavioral problems for the positive effects that could produce the 5-HT synthesis [32]. 

However, the adoption of a dissociate diet, although probably easily accepted by the owners, requires some caveats. The diet could cause a hyperglycemic peak if a rapid insulin incretion is lacking. Therefore, careful monitoring of the dog glycaemia should be carried out to avoid treating animals suffering from diabetes or inducing it with a long-term administration of a dissociated diet. It could be interesting to evaluate the effects of different kinds of carbohydrates, with different glycemic index.

Recent research has also highlighted that hyperglycemia can initiate proinflammatory events, followed by an overproduction of reactive oxygen species and inhibition of endothelial dependent nitric oxide synthase [33], which may impair endothelial function. For these reasons, further research is needed to clarify whether a dissociated diet should be implemented as a matter of course in a veterinary practice. However, in case its use is advised, it should be prescribed for a limited period, under the strict supervision of a veterinary surgeon, along with a behavioral modification therapy.

## 5. Conclusions

In this study, a carbohydrate morning meal, compared to other meals, was found to lead to significantly higher TRP/5LNAAs ratios for at least 6 h. It is possible that the higher bioavailability of TRP, and the consequent increase of 5-HT in the brain may have positive effects on dog behavioral problems, characterized by a 5-HT deficit. Nevertheless, further studies will be required to understand if the high TRP/5LNAAs ratio, obtained with this type of meal, will be sufficient to guarantee a gain of 5-HT synthesis and, consequently, result in an improvement of behavior. In addition, future research should clarify how to deal with possible side effects of a dissociated diet, such as the potential hyperglycaemia after the carbohydrates meal, and the potential problems of TRP/5LNAAs ratio reduction after the meat-based meal.

## Figures and Tables

**Table 1:** Food and daily diet chemical composition (% on Dry Matter basis). D1: homemade diets based on puffed rice, beef meat and olive oil. D2: commercial dry food for adult dogs.

	Beef Meat	Puffed Rice	D1	D2
Dry Matter	33.20	94.40	44.56	92.00
Crude protein	59.34	6.57	27.74	28.26
Crude fat	39.15	1.91	16.78	16.30
NSC	-	73.83	46.40	46.64
Crude fiber	-	1.38	0.87	2.50
Ash	1.51	16.31	8.21	6.30
ME (kcal/kg)	5405	2976	4021	4007

**Table 2:** Amount of administered food for 30 kg BW (as fed basis). M1 = Meal 1; M2 = Meal 2; M3 = Meal 3.

	M1		M2		M3	
	8:00 AM	8:00 PM	8:00 AM	8:00 PM	8:00 AM	8:00 PM
Puffed Rice (g)	125	125	250	-	-	-
Beef Meat (g)	225	225	-	450	-	-
Olive Oil (g)	3	-	3	-	-	-
Dry food (g)	-	-	-	-	260	260

**Table 3:** TRP/5LNAAs ratio values (mean ± S.D.) in the three meals at the different times. M1 = Meal 1; M2 = Meal 2; M3 = Meal 3.

Time	M1	M2	M3	*p*
t0	0.217 ± 0.037	0.229 ± 0.031	0.213 ± 0.025	n.s.
t2	0.224 ± 0.030 ^b^	0.306 ± 0.049 ^a^	0.217 ± 0.035 ^b^	0.001
t4	0.225 ± 0.026 ^b^	0.327 ± 0.058 ^a^	0.197 ± 0.036 ^b^	0.001
t6	0.244 ± 0.030 ^b^	0.303 ± 0.049 ^a^	0.205 ± 0.030 ^b^	0.015
t8	0.246 ± 0.020 ^ab^	0.280 ± 0.039 ^a^	0.206 ± 0.025 ^b^	0.001
t10	0.243 ± 0.029 ^a^	0.294 ± 0.041 ^a^	0.224 ± 0.017 ^b^	0.001
t24	0.210 ± 0.024 ^a^	0.155 ± 0.027 ^b^	0.189 ± 0.020 ^a^	0.041

^a,b^: Means significant differences between columns. n.s.: Not statistically significant.
